# Psychological assistance to adolescents after spinal cord injury

**DOI:** 10.1192/j.eurpsy.2025.905

**Published:** 2025-08-26

**Authors:** Y. Sidneva, E. Lvova, S. Valiullina

**Affiliations:** 1 rehabilitation, Clinical and Research Institute of Emergency Pediatric Surgery and Trauma; 2 neuropsychiatric, N.N. Burdenko Neurosurgery Institute, Moscow, Russian Federation

## Abstract

**Introduction:**

Spinal cord injury (SCI) is a severe trauma that leads to disability and decreased social activity. Recently, the number of patients among children has been growing. In Russia, the share of spinal cord injury in the structure of all injuries is 18.3%.

Post-traumatic consequences include neurological, motor and somatic disorders, as well as psychological ones. Psychological support for children with severe SCI is an important link in comprehensive rehabilitation at all its stages. There are data in the literature on the psychological state after spinal cord injuries, but this is in adults.

**Objectives:**

to study psychological problems in adolescents after severe spinal cord injury in the early stages of rehabilitation.

**Methods:**

50 patients (12-18 years old) admitted for treatment and rehabilitation on the 1st-3rd day after spinal cord injury. Psychological diagnostics included: clinical interview; Anxiety and Personality Disorders Inventory (Spielberger 1983, adapted by Khanin); Beck Depression Inventory (Beck 1961, version for adolescents); Recovery Locus of Control (Partridge, Johnston 1989).

**Results:**

All patients (100%) had psychological problems of varying severity: 80% of adolescents - high level of reactive anxiety; 28.3% - low motivation for rehabilitation; 33.3% - depression (of which 30% - reactive depression; 3.3% - masked depression). Symptoms could be combined and observed in the same patient.

Children with high levels of personal anxiety in combination with reduced levels of motivation had difficulties in adaptation to rehabilitation.

**Conclusions:**

All patients (100%) require psychological support in order to correct emotional background and motivation. Differentiated specialized assistance, including psychological, will increase the effectiveness of rehabilitation. This will help to adapt adolescents with post-traumatic deficiency to the changed conditions of life, improve its quality and return them to their previous social environment.

**Disclosure of Interest:**

None Declared

